# Virus-Receptor Mediated Transduction of Dendritic Cells by Lentiviruses Enveloped with Glycoproteins Derived from Semliki Forest Virus

**DOI:** 10.1371/journal.pone.0021491

**Published:** 2011-06-27

**Authors:** Steven Froelich, April Tai, Katie Kennedy, Adnan Zubair, Pin Wang

**Affiliations:** 1 Mork Family Department of Chemical Engineering and Materials Science, University of Southern California, Los Angeles, California, United States of America; 2 Department of Biomedical Engineering, University of Southern California, Los Angeles, California, United States of America; 3 Department of Pharmacology and Pharmaceutical Sciences, University of Southern California, Los Angeles, California, United States of America; Agency for Science, Technology and Research - Singapore Immunology Network, Singapore

## Abstract

Lentiviruses have recently attracted considerable interest for their potential as a genetic modification tool for dendritic cells (DCs). In this study, we explore the ability of lentiviruses enveloped with alphaviral envelope glycoproteins derived from Semliki Forest virus (SFV) to mediate transduction of DCs. We found that SFV glycoprotein (SFV-G)-pseudotyped lentiviruses use C-type lectins (DC-SIGN and L-SIGN) as attachment factors for transduction of DCs. Importantly, SFV-G pseudotypes appear to have enhanced transduction towards C-type lectin-expressing cells when produced under conditions limiting glycosylation to simple high-mannose, *N*-linked glycans. These results, in addition to the natural DC tropism of SFV-G, offer evidence to support the use of SFV-G-bearing lentiviruses to genetically modify DCs for the study of DC biology and DC-based immunotherapy.

## Introduction

The fundamental rationale behind gene-based immunotherapy lies in the ability to modify immune cells to achieve a therapeutic benefit. We and others have developed methods to target gene delivery to specific immune cell types [1]. Recent realization that antigen presenting cells (APCs) are powerful tools for the manipulation of the immune system, has led to new targets for gene-based immunotherapy. The application of gene delivery for immunization relies on a new strategy in which dendritic cells (DCs), the most powerful APCs which can initiate and maintain immune responses by stimulating both T and B cells, are genetically modified to express antigens or produce immunostimulatory molecules to create a therapeutic advantage [2].

Efficient gene-based immunization has been achieved using a variety of viral vectors, each of which have specific advantages and drawbacks [3]. Lentiviruses have emerged as a particularly efficient and promising tool for gene transfer into a wide range of immune cell types. They have been shown to be very effective in delivering genes into DCs [3], [4], [5], [6], [7], [8]. DCs that are transduced by antigen-encoding lentiviruses are able to efficiently present the antigens and stimulate antigen-specific responses either *in vitro* [9], [10], [11], [12], or after *in vivo* transplantation [9], [13], [14], [15], and even through direct injection of the vector *in vivo* [9], [16], [17], [18].

One strategy for directing the cellular transduction is through pseudotyping lentiviruses with glycoproteins from other enveloped viruses which have a natural tropism for APCs [19]. Recently, the mosquito-produced Sindbis alphavirus [20] and lentiviruses with engineered Sindbis glycoproteins [21] were shown to use C-type lectins as attachment receptors leading to productive transduction of DCs *in vivo*. The C-type lectins DC-SIGN and L-SIGN (together known as DC-SIGN(R) [22]) are important targets because they are promiscuous receptors capable of capturing viruses on APCs located throughout the body [23], [24]. Both lectins are tetrameric type II transmembrane proteins composed of a carbohydrate recognition domain (CRD), which binds to high-mannose oligosaccharides in a calcium-dependent manner, and a short cytoplasmic tail responsible for signaling and internalization that can activate APCs leading to an immune response to the viral vectors or their transgene products [25], [26], [27], [28].

Pseudotyped recombinant lentiviruses using the Semiliki Forest (SFV) envelope glycoproteins have recently been reported [29], [30]. These pseudotypes are particularly promising because they are able to be concentrated to obtain high-titer viral preparations, resistant to inactivation by components in human sera, and stable packaging cell lines have previously been generated that could be scaled up to meet the larger volumes for clinical demand [30]. In this study, we generated recombinant lentiviral particles bearing envelope glycoproteins of SFV (SFV-G) and investigated the ability SFV-G to facilitate transduction of DCs. In light of previous findings with other alphaviral envelope glycoproteins, we investigated the role of C-type lectins to act as attachment factors for SFV-G. Our current studies identified effects of DC-SIGN and L-SIGN on binding and transduction for SFV-G-pseudotyped lentiviruses. Our data evaluating these pseudotyped viral particles suggests that SFV-G facilitates binding and transduction through C-type lectins which is enhanced when produced under high mannose N-glycan glycosylation conditions.

## Results

### DC-SIGN correlates with increased infection of lentivirus pseudotyped with SFV-G

**Figure 1:**
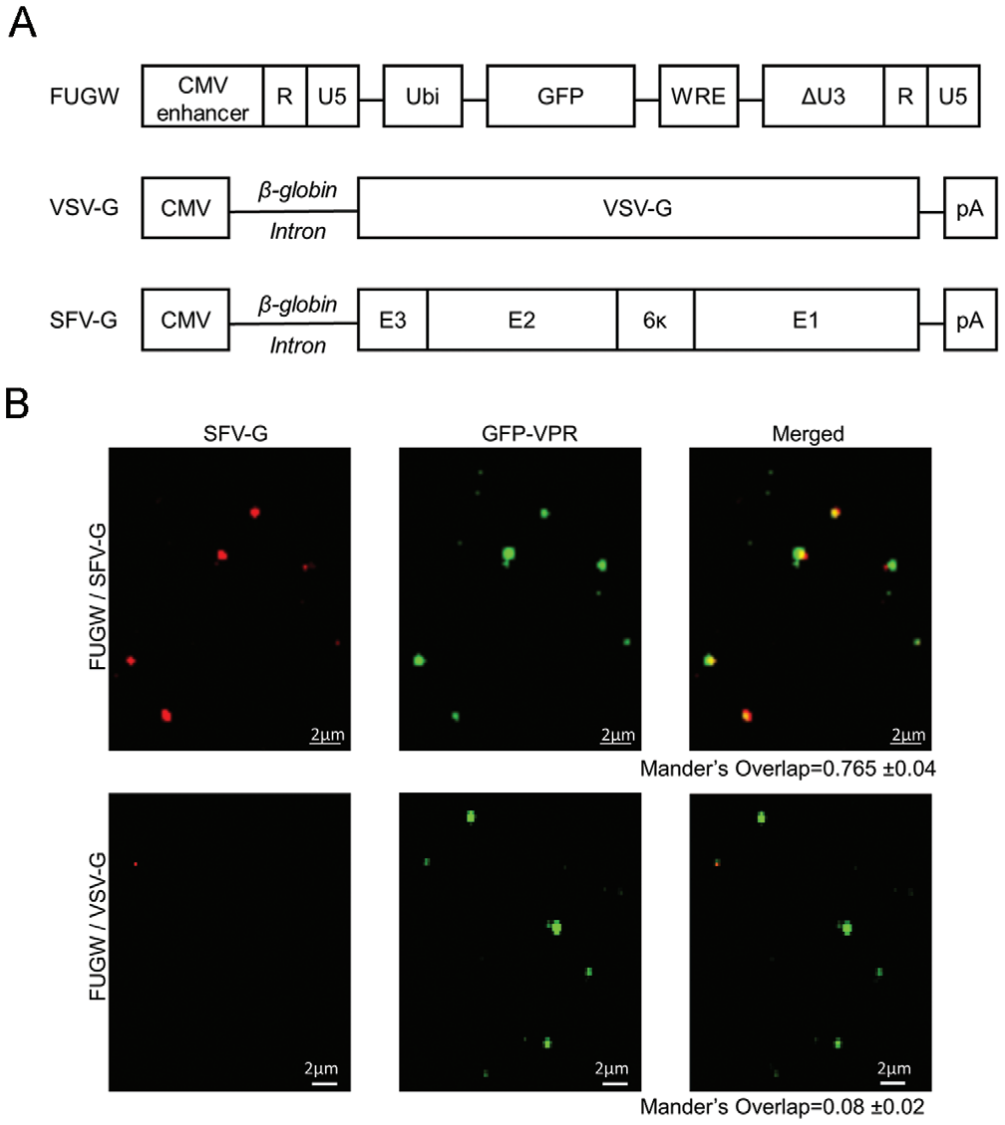
Virus-producing constructs used to make pseudotyped lentiviruses. (A) Schematic diagrams of constructs encoding the lentiviral backbone FUGW and envelope glycoproteins. Ubi: the human ubiquitin-C promoter; GFP: enhanced green fluorescence protein; WRE: the woodchuck hepatitis virus posttranscriptional regulatory element (WRE) to increase the level of transcription; ΔU3: deleted U3 region that results in the transcriptional activation of the integrated viral LTR promoter; pA: polyadenylation signal; E1, E2, 6k, E3: SFV-glycoprotein (E1 for fusion, E2 for receptor binding, 6k a linker, and E3 is a signal sequence). The VSV-G expressing plasmid contains the rabbit β-globin intron and poly(A) signal. (B) Viral supernatants harvested from virus-producing cells transiently transfected with GFP-vpr, SFV-G, or VSV-G, and other necessary packaging constructs, were coated to a poly-lysine containing coverslip by centrifugation. The resulting coverslips were then rinsed and immunostained with an anti-SFV-G antibody (red) to label the glycoproteins and imaged using a laser confocal microscope.

It has been shown that SFV-G can pseudotype HIV-1-derived lentiviruses [29], [30]. We standardized the constructs for expressing envelope proteins by sub-cloning the SFV-G expression cassettes into the VSV-G expression vector containing the rabbit β-globin intron and the poly(A) signal sequence (Figure 1A); the plasmid used for VSV-G expression has been used extensively for pseudotyping lentiviruses [29]. Lentiviruses pseudotyped with alphaviral glycoproteins were generated by the co-transfection of 293T cells with the lentiviral construct FUGW, plasmids encoding viral *gag*, *pol*, and *rev* genes, and the envelope protein expression plasmid. FUGW carries the GFP reporter gene under the control of the human ubiquitin-C promoter (Figure 1A) [31]. GFP-vpr-labeled lentiviruses were produced as described above with an additional plasmid encoding the GFP protein fused to the HIV-1 *vpr* protein [32]. Antibody staining with anti-SFV-G of GFP-vpr-labeled virus revealed significant overlap (Mander's overlap coefficient >0.7) for SFV-G- but not VSV-G (Mander's overlap coefficient <0.1) pseudotyped viruses (Figure 1B). Differences in particle sizes were observed and could be due to objects being on a slightly different focal plane or the presence of non-functional viral like particles. These results indicate that SFV-G is incorporated onto lentiviral particles.

**Figure 2:**
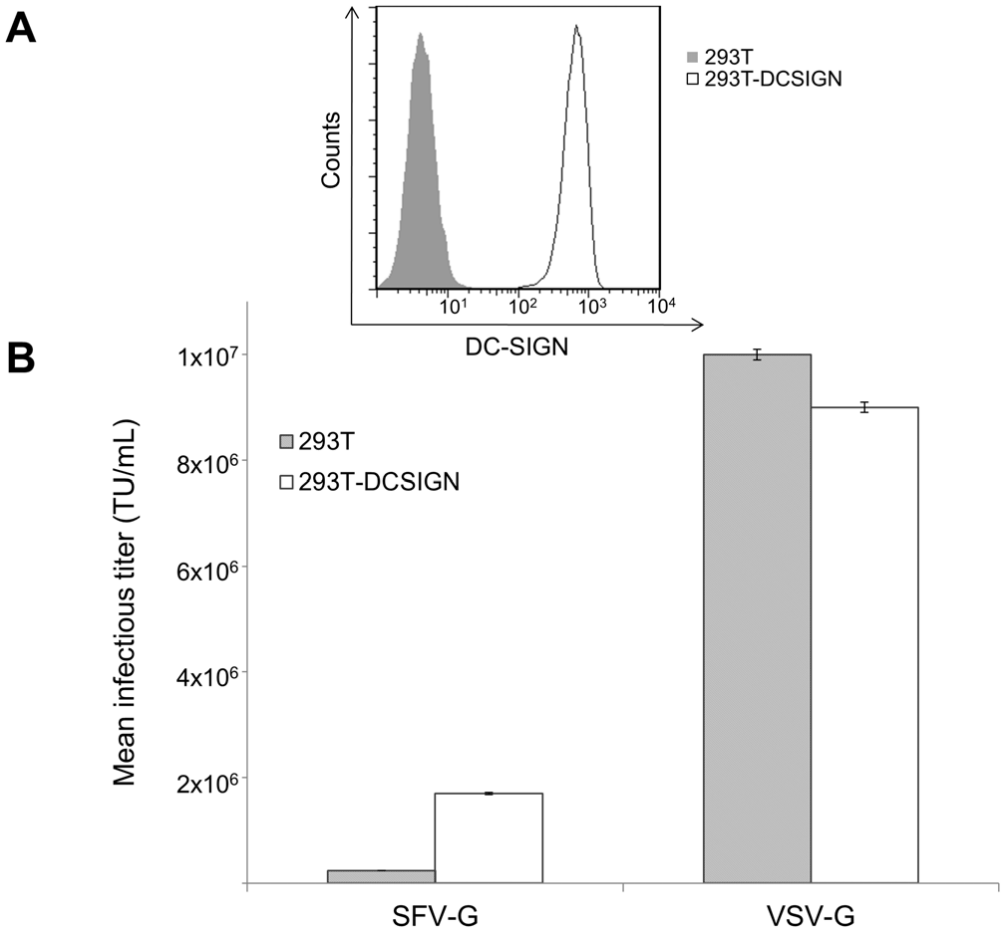
Lentiviral transduction of DC-SIGN-expressing 293T cells. (A) Expression of DC-SIGN in 293T (solid fill) and 293T.DCSIGN (open fill) was detected by flow cytometry. (B) Transduction titer of lentiviruses were quantified by serially diluting fresh viral supernatants of FUGW/SFVG and FUGW/VSVG and used to transduce 2×10^4^ 293T.DC-SIGN (open bars) or parental 293T cells (solid bars). Three days later, the transduction efficiency was measured by analyzing GFP expression using flow cytometry where the corresponding viral titer was calculated. Values are given as the mean of triplicates ± S.E.

To characterize the infectivity of SFV-G- and VSV-G-bearing lentiviruses, the infectious titer was measured on parental 293T cells and the 293T.DCSIGN cell line which stably expresses human DC-SIGN (Figure 2A). Differences in viral titer may be attributed to several factors: differences in virus-receptor interactions, the efficiency of production of functional particles into the supernatant, as well as the amount of defective particles which may serve as interfering particles. Consistent with previous reports [29], [30], SFV-G and VSV-G can both pseudotype lentivirus to produce infectious particles; these viruses are designated FUGW/SFVG and FUGW/VSVG, respectively. When 293T or 293T.DCSIGN cells were transduced with serially diluted viral supernatants the titer of the VSV-G-pseudotyped lentivirus (FUGW/VSVG) was calculated to be approximately 10×10^6^ transduction units (TU)/mL for both cell types. When SFV-G was used as the envelope glycoprotein, the infectious titer based on 293T a cell was ∼40 times lower than VSV-G (Figure 2B). However, the SFV-G-bearing lentivirus was much more infectious for 293T.DC-SIGN cells; the titer was about 7-fold higher than measured on 293T cells. The difference in infectious units between cell types is clear evidence that the transduction of SFV-G-bearing viruses is enhanced by the presence of DC-SIGN.

### Preferential transduction of DC-SIGN- or L-SIGN-expressing 3T3 cells

**Figure 3:**
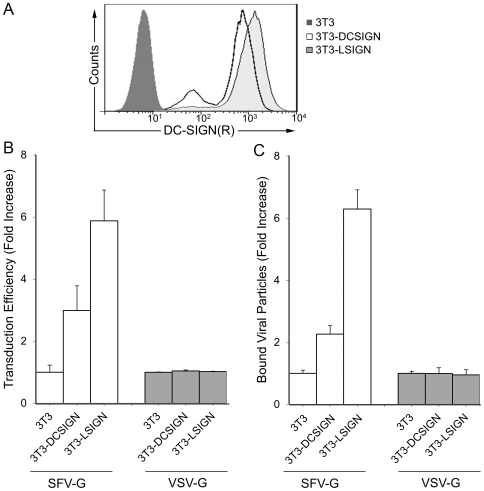
Effects of DC-SIGN or L-SIGN expression. (A) Expression of L-SIGN and DC-SIGN in 3T3 (solid fill), 3T3.DCSIGN (open fill) and 3T3.LSIGN (gray fill), respectively, was detected using cross reactive DC-SIGN/L-SIGN antibody and quantified by flow cytometry. (B) Effects of DC-SIGN or L-SIGN expression on the infectivity of pseudotyped lentiviruses. SFV-G- and VSV-G-pseudotyped lentiviruses were normalized by p24 and spin-inoculated with LSIGN- or DCSIGN-expressing 3T3 cells; the parental 3T3 cells were included as controls. Three days later, the transduction efficiency was measured by analyzing GFP expression. Fold increase in percentage of GFP-positive cells is shown based on 3T3 cells where FUGW/SFVG transduced 6.6±0.7% and FUGW/VSVG transduced 72.2±1.0%. (C) Specificity of binding to DC-SIGN. [^35^S]-methionine-labeled FUGW/SFVG or FUGW/VSVG were incubated with 3T3 or DC-SIGN/L-SIGN-expressing cells at 4°C. Cells were washed and ^35^S radioactivity of the resuspended cells was quantitated with a liquid scintillation counter. Fold increase in [^35^S] bound viral particles is shown based on 3T3 cells with 3.59±0.27% and 25.23±1.25% of the total CPM of virus bound for FUGW/SFVG and FUGW/VSVG, respectively, where values are given as the mean of triplicates ± S.E.

Previous studies have indicated that the cell type in which DC-SIGN(R) is expressed can have a significant impact on the efficiency of these lectins to promote viral infection [33], [34]. To study in alternative cell types the function of C-type lectins as attachment factors, we transduced the 3T3-L-SIGN and 3T3-DC-SIGN cell lines and the corresponding parental 3T3 cells. The DC-SIGN or L-SIGN expression on these cell lines was confirmed using a cross reactive DC-SIGN/L-SIGN monoclonal antibody (Figure 3A). These cell lines were transduced by SFV-G- and VSV-G-bearing lentiviruses. After 48 hrs, cells were analyzed by flow cytometry for GFP expression. The levels of transduction were normalized based on 3T3 transduction, and the magnitude of the increase in transduction was assessed (Figure 3B). SFV-G-pseudotyped virus showed a preferential increase in transduction with cells expressing L-SIGN (6 folds) and DC-SIGN (3 folds), whereas VSV-G-bearing particles did not exhibit a significant preference (Figure 3B).

To determine if the increase in transduction of pseudotyped lentiviruses for 3T3 cells expressing DC-SIGN or L-SIGN was due to greater cell binding, *in vitro* attachment assays were performed with [^35^S]-methionine-radiolabeled virus. Results of assays with SFV-G-bearing particles showed a direct correlation between an increase in percentage of virus bound and the observed increase in infectivity (Figure 3C). The SFV-G-bearing particles bound 2- to 6-fold more efficiently to DC/L-SIGN-expressing cells than to 3T3 cells. Consistent with the infection data, the level of increase of binding to DC-SIGN-expressing cells was generally lower than that to L-SIGN-expressing cells. In the absence of DC-SIGN expression, SFV-G pseudotypes bound poorly to 3T3 cells with only 2,289 CPM binding to cells out of the 63,701 CPM that were incubated with the cells. VSV-G-pseudotyped virus exhibited similar levels of attachment to all cell types (Figure 3C) with approximately 25% of the total CPM of virus bound to all cell types. As shown in Figure 3B and 3C, the attachment and transduction of FUGW/VSVG was approximately similar among cells regardless of DC-SIGN(R) expression. This is consistent with the lack of increased infectivity observed with FUGW/VSVG on DC-SIGN expressing cells. Our data suggest that SFV-G-bearing lentiviruses utilize DC-SIGN and L-SIGN as attachment receptors to facilitate binding and transduction.

### SFV-G- and VSV-G-mediated transduction requires acidification

**Figure 4:**
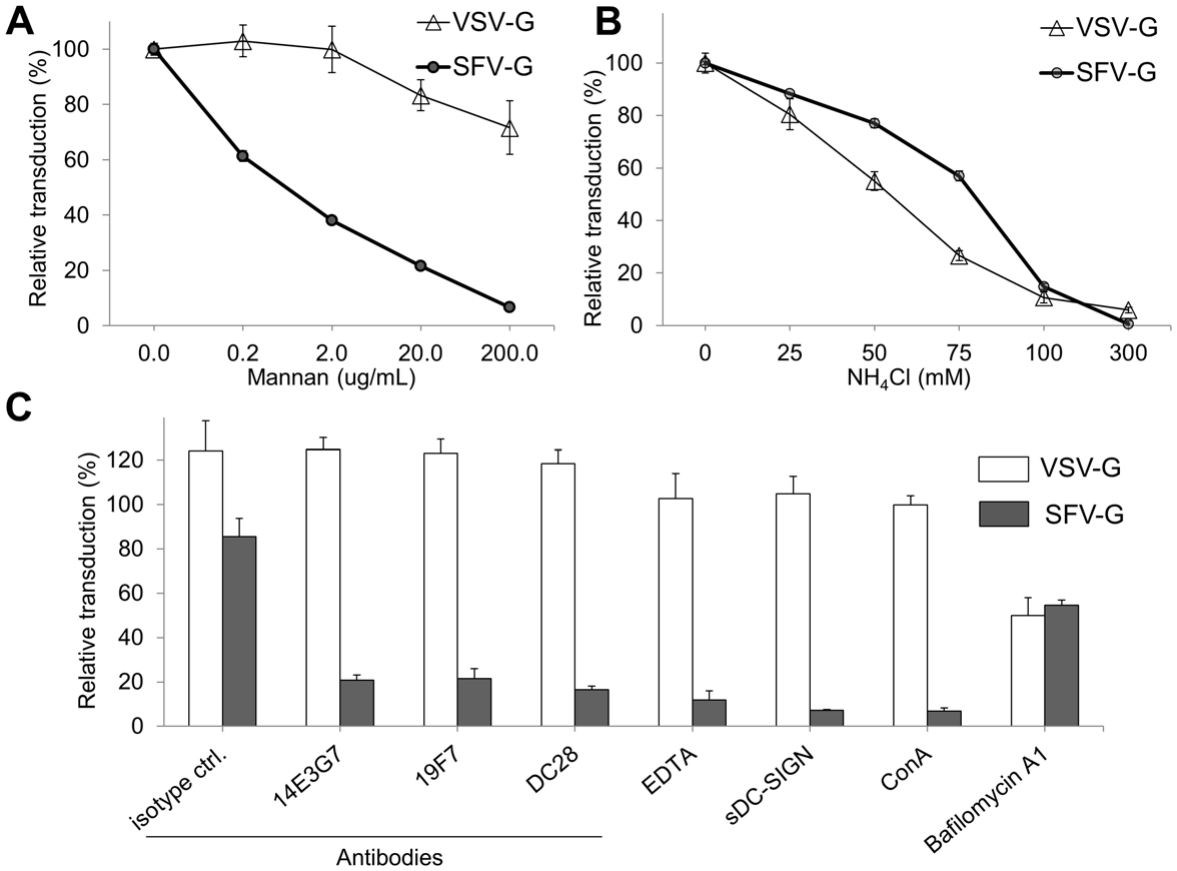
Specific inhibitors prevent DC-SIGN-mediated infection. In dose-response experiments, 293T.DCSIGN cells were treated with SFV-G (solid circles) or VSV-G (open triangles) lentiviruses in the presence of increasing concentrations of mannan (A), or NH_4_Cl (B). (C) 293T.DCSIGN cells were incubated with antibodies at a concentration of 5 µg/ml, 15 nM Bafilomycin A_1_, 5 mM EDTA, or 10 µg/ml soluble DC-SIGN at 37°C for 30 min, and then inoculated with SFV-G (filled bars) or VSV-G (open bars) lentiviruses at an MOI∼0.8 for 8 hrs or lentiviruses incubated with 25 µg/mL ConA (1 h at 37°C). Subsequently, the supernatants were replaced and incubated with fresh medium for two days before being analyzed for GFP expression. The relative transduction was determined based on non-treated controls and values are given as the mean of triplicates ± S.E.

Internalization of SFV-G and VSV-G lentiviruses is known to involve the endocytic pathway, in which the acidic environment of endosomal vesicles is required. Treatment with the pH-interfering drugs ammonium chloride caused a dose-dependent reduction of infectivity in 293T.DCSIGN cells for both viruses (Figure 4B). This suggests that both DC-SIGN-mediated infection of FUGW/SFVG and DC-SIGN-independent infection by FUGW/VSVG are pH-dependent processes, presumably due to the acidification requirement of the virus-endosome fusion process [32]. Transduction was inhibited by endosomal neutralization using bafilomycin A_1_ (Figure 4C), verifying pH-mediated transduction with both SFV- and VSV-G bearing particles.

### SFV-G-mediated infectivity can be blocked with inhibitors of DC-SIGN

We observed that the infection efficiency of SFV-G-bearing lentiviruses correlates with the expression of DC-SIGN on target cells. To further examine the specificity of virus interaction with these molecules, we performed infectivity experiments in the presence of increasing concentrations of yeast mannan, or ethylenediaminetetraacetic acid (EDTA), soluble recombinant DC-SIGN, anti-DC-SIGN monoclonal antibodies (mAbs), or an isotype-matched control (Figure 4A and 4C). These treatments disrupt interactions with DC-SIGN molecules [20], [22], [26]. Incubation with mannan, a carbohydrate that competitively inhibits virus binding with DC-SIGN [35], during viral inoculation of FUGW/SFVG with 293T.DCSIGN cells resulted in a dose-dependent reduction in the amount of GFP-positive cells (Figure 4B). However, mannan was not as effective at inhibition of VSV-G-bearing lentivirus, suggesting a different receptor interaction between VSV-G and 293T.DCSIGN cells. The principal characteristic of C-type lectins is that they interact with mannose residues of viral glycoproteins in a calcium-dependent manner via their C-terminal carbohydrate recognition domain (CRD). EDTA is a calcium chelator and treatment during infection with FUGW/SFVG resulted in a >70% reduction in GFP-positive cells (Figure 4C). In contrast, FUGW/VSVG virus was not dependent on calcium for infection as revealed by the EDTA inhibition experiment. Incubation with anti-DC-SIGN mAbs during infection also resulted in a reduction in the numbers of GFP-positive cells with FUGW/SFVG infection, whereas the isotype control antibody had little inhibitory effect (Figure 4C). Again, the FUGW/VSVG virus did not exhibit significant inhibition in the presence of DC-SIGN antibodies (Figure 4C). Furthermore, pre-incubation of FUGW/SFVG lentivirus with concavalin A (ConA), which binds to *N*-linked high-mannose structures reduced infectivity by ∼90% (Figure 4C). The inhibition by ConA supports the theory that mannose carbohydrate residues present on SFV-G participate in the attachment to DC-SIGN. Finally, infection of SFV-G- but not VSV-G-bearing virus was blocked by pre-incubation of the cells with soluble DC-SIGN (Figure 4C). The results of these experiments indicate that the infectivity of SFV-G-pseudotyped lentivirus is dependent on DC-SIGN expression for transduction, whereas FUGW/VSVG transduction is not dependent upon DC-SIGN.

**Figure 5:**
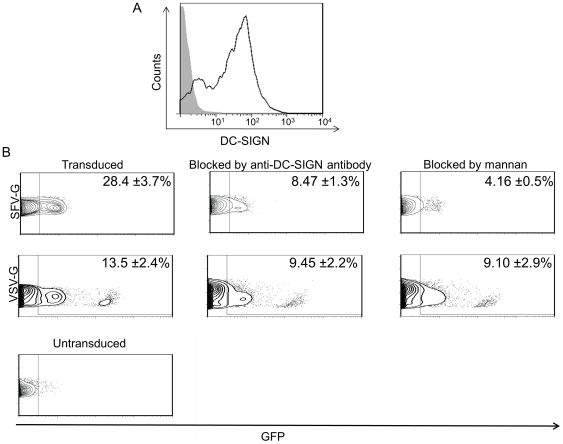
Transduction of MoDCs by lentiviruses is inhibited with anti-DCSIGN antibody and mannan. Human monocyte-derived DCs (MoDCs) were generated by culturing respective precursor cells in the presence of GM-CSF and IL-4. (A) The adherent cells (1×10^6^) were cultured for 2 days and then DC-SIGN expression was detected by flow cytometry. (B) Human MoDCs (1×10^6^) were incubated for 1 hour with mannan (200 µg/mL), anti-DC-SIGN(R) antibody (20 µg/mL) or without any reagents. The cells were then infected with FUGW/SFVG or FUGW/VSVG (MOI = 10) for 8 hours in the presence of blocking reagents. GFP expression was assayed by flow cytometry five days post-transduction where one representative figure is shown with values given as the mean of triplicates ± S.E.

We further evaluated the ability of inhibitors of DC-SIGN to block infection of human monocyte derived DCs (MoDCs). MoDCs were prepared from the peripheral blood mononuclear cells (PBMC) of healthy human donors and cultured with GM-CSF and IL-4 to generate DC-SIGN^+^ DCs [36]. More than 80% of the cultured DCs were positive for DC-SIGN expression before infection (Figure 5A). MoDCs were challenged with the same MOI of SFV-G- and VSV-G-pseudotyped lentiviruses incubated in the presence of mannan or anti-DC-SIGN(R) antibody. Transduction of MoDCs by FUGW/SFVG decreased from ∼28% to ∼8% with anti-DC-SIGN antibody and to ∼4% in the presence of yeast mannan (Figure 5B). The transduction efficiency of FUGW/VSVG was lower than that of the SFV-G bearing lentivirus (∼13% GFP^+^). In contrast to FUGW/SFVG, there was not a significant decrease when FUGW/VSVG was incubated with MoDCs in the presence of either mannan or anti-DC-SIGN(R) antibody (Figure 5B). These results suggest that DC-SIGN(R) function as a SFV-G binding molecule that is required for the productive infection of MoDCs.

### Effects of viral glycosylation

**Figure 6:**
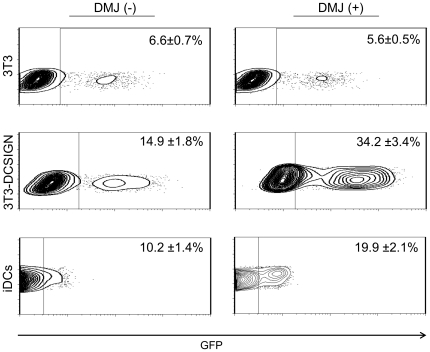
Transduction by SFV-G lentiviruses produced in DMJ treated cells. 3T3 (2×10^4^), 3T3-DCSIGN (2×10^4^) and MoDCs (1×10^6^) were spin-infected with FUGW/SFVG produced in 293T cells without DMJ(−) or with DMJ(+) treatment. GFP expression was assayed by flow cytometry three days post-transduction where values are given as the mean of triplicates ± S.E.

Next, we evaluated the ability mannose carbohydrate structures on the SFV-G lentiviral particles to promote infection through a mechanism of increase interactions with DC-SIGN(R). To test this, we generated FUGW/SFVG particles containing only high-mannose glycan content on their envelope glycoproteins by treating virus-producing cells with 1-deoxymannojirimycin (DMJ). DMJ is an inhibitor of Golgi mannosidase I and can arrest glycan maturation primarily at the Man_8_GlcNAc_2_ stage [37]. Production of pseudotyped lentiviruses in the presence of DMJ altered their ability to transduce DC-SIGN-expressing 3T3 cells (Figure 6, DMJ (+)). A two fold increase in transduction efficiency of 3T3-DCSIGN cells was observed for DMJ-treated SFV-G-bearing virus, whereas no significant transduction increase in parental 3T3 was observed. We further tested how efficiently SFV-G pseudotyped lentiviruses transduce primary immune cell targets, MoDCs, and the effect of DMJ treatment on transduction efficiency. Transduction of MoDCs by DMJ-treated FUGW/SFVG was approximately twice as efficient as non-treated FUGW/SFVG, transducing ∼20% versus ∼10% MoDCs (Figure 6). Similar to the 3T3-DCSIGN cell line, transduction by SFV-G pseudotyped lentiviruses produced in the presence of DMJ increased their ability to transduce MoDCs (Figure 6). Together, these results reveal that the 293T-produced SFV-G-bearing particles are able to innately bind to DC-SIGN(R)-receptors but DMJ-treatment can enhance transduction of DC-SIGN-expressing cells.

## Discussion

We have demonstrated that SFV-G-bearing lentiviruses can utilize DC-SIGN and L-SIGN as attachment receptors, resulting in productive infections of cell lines bearing these molecules and human MoDCs. Preferential binding to DC-SIGN(R) by SFV-G has not previously been reported, indicating that there is an unappreciated naive trophism of SFV-G pseudotyped lentivirus for APCs. Our results suggest that by utilizing the affinity of SFV-G to DC-SIGN(R), lentiviruses can be engineered to preferentially transduce antigen-presenting DCs for gene-based immunotherapy.

We found that FUGW/SFVG but not FUGW/VSVG produced in 293T cells bind to DC-SIGN(R) receptors. When produced in 293T cells, VSV-G-pseudotyped lentiviruses exhibited similar binding and transduction of cells expressing DC-SIGN, L-SIGN or parental cell lines. For SFV-G-bearing viruses, cells expressing DC-SIGN or L-SIGN were more permissive than non-expressing parental cell lines. The increased transduction was well-correlated with an increase in binding to the cells as measured by radiolabeled virus binding assays. Specific interaction between DC-SIGN and SFV-G was demonstrated by blocking the transduction of the DC-SIGN-expressing cells with inhibitors such as ConA, EDTA, soluble DC-SIGN protein, yeast mannan, or DC-SIGN-specific mAbs in both 293T.DCSIGN cells and human MoDCs.

SFV-G-bearing lentiviruses have an enhanced ability to transduce DC-SIGN-expressing cells when produced under conditions arresting the viral glycan maturation primarily at the high mannose stage. Several reports indicate that the presence of high-mannose-content *N*-linked glycans on Sindbis virus, Ebola virus and West Nile virus enhance the infection of mouse-derived DCs due to interactions with the mannose binding C-type lectin receptors [20], [22], [38]. In addition to DC-SIGN present on DCs, there are other C-type lectin molecules on macrophages, endothelial cells, and other APCs, that might play a role in FUGW/SFV-G transduction [23], [25]. High mannose content of viral envelope glycoproteins directly influences the efficiency of viral capture by DC-SIGN and L-SIGN [20], [39], [40]. Similar to findings reported previously for Sindbis virus [41], when SFV-G-bearing lentiviruses were generated in mammalian cells treated with the mannosidase I inhibitor DMJ, the resulting particles exhibit an increased capacity to utilize DC-SIGN for infection. The increase in interaction with DC-SIGN by FUGW/SFVG is presumably mediated by increased binding by the CRD to the mannose structures of these glycoproteins. The SFV-G has four sites for *N*-linked glycosylation (E1-141, E2-200, E2-262 and E3-14) [42]. The E3 protein is cleaved from the mature SFV particles, but remains associated with the virion of SFV [43]. When virus-producing cells are treated with DMJ, transduction by SFV-G-bearing virus was increased towards MoDCs as well as DC-SIGN-expressing 3T3 cells but not the parental 3T3 cell line. The observation that FUGW/SFVG produced by DMJ-treated cells have an enhanced ability to preferentially transduce DC-SIGN-expressing cells suggests that modification of *N*-linked glycans of SFV-G can be used to enhance the transduction of DCs.

We observed that FUGW/VSVG lentiviruses exhibited no increase in binding or transduction with cells expressing DC-SIGN or L-SIGN. VSV-G-pseudotyped viruses do not target through DC-SIGN(R) [44], [45] unless produced with in the presence of DMJ [46] but they also efficiently transduce a wide range of cell types, likely through a ubiquitous membrane lipid [47]. Although previous studies have found that VSV-G pseudotypes of HIV-1 infect bone marrow-derived premature DCs [30], we found that FUGW/VSVG exhibited no preferential transduction towards cells expressing the human C-type lectins DC-SIGN or L-SIGN. FUGW/VSVG lentiviruses have a broad tropism and can transduce multiple cell types. Therefore, they are undesirable for delivering genes *in vivo* to APCs. Our results suggest that the transduction by FUGW/VSVG is largely DC-SIGN(R)-independent and less efficient than FUGW/SFVG at transducing DCs when normalized by infectious particles.

In this study we assessed the relationship of DC-SIGN and L-SIGN interactions with SFV glycoproteins to mediate the transduction of DCs. DCs are potent APCs and play a major role in the activation of both memory and naïve T cells. Genetically modified DCs have been used to elicit antigen-specific, major histocompatibility complex-restricted cytotoxic T lymphocyte (CTL) responses [21], [48]. The development of DC differentiation protocols for PBMC has facilitated the study of DC biology and the subsequent implementation of clinical DC-based vaccination studies. Transduction of human MoDC by FUGW/SFVG was more than twice as efficient as that by FUGW/VSVG when normalized by MOI. Furthermore, the transduction by FUGW/SFVG was DC-SIGN(R)-specific and could be inhibited by both yeast mannan and anti-DC-SIGN antibodies. SFV-G pseudotypes preferentially transduced the DC-SIGN-positive cells, consistent with the theory that DC-SIGN mediates transduction in DCs. The preferential transduction of DCs can be further enhanced by production under untrimmed (DMJ-treated) high mannose conditions. Further studies to assess the maturation of DCs transduced by FUGW/SFVG vectors by measuring maturation markers such as HLA-DR, CD11b, and CD83 and the effect on type I interferon production in DCs are ongoing. FUGW/SFVG has previously been shown to have rather broad tropism and be able to have low transduction efficiency for a wide range of cell types [30], but we have found that FUGW/SFVG has a DC-SIGN(R) tropism, which can be utilized for directing the cellular transduction of lentiviruses to APCs.

The results described herein have relevance to the design and production of viral vectors used for gene delivery to APCs. Targeting of lentiviruses to C-type lectin-expressing cells such as DCs can be increased by pseudotyping with the Semliki Forest virus envelope glycoprotein and further enhanced by production under conditions that limit host cell processing of viral carbohydrate modifications to contain mannose structures. When lentiviruses were produced in DMJ-treated cells, they generated a similar amount of physical viral particles as those produced in normal conditions (unpublished data). Enhanced delivery of antigen to immature DCs may provide an opportunity for improvement of gene-based vaccination approaches. Future studies are warranted to investigate whether the wild type Semliki Forest virus has a similar tropism for DC-SIGN(R) expressing cells. Our results show that SFV-G pseudotyped lentivectors strongly bind to C-type lectins. The affinity of FUGW/SFVG with DC-SIGN(R) represents a new strategy to genetically modify DCs.

## Materials and Methods

### Cell lines

293T.DCSIGN were derived as previously described [21] and stained (anti-DC-SIGN antibody from BD Biosciences) to confirm expression of DC-SIGN. Mouse fibroblasts NIH 3T3 cells were obtained from the American Tissue Culture Collection (ATCC, Manassas, VA). 3T3-L-SIGN and 3T3-DC-SIGN were obtained from the NIH AIDS Research and Reference Reagent Program, Division of AIDS, NIAID. These cell lines were maintained in DMEM medium (Invitrogen, Carlsbad, CA) supplemented with 10% fetal calf serum (Sigma-Aldrich, St. Louis, MO), 2 mM L-glutamine, and 100 U/mL of penicillin and 100 µg/mL of streptomycin.

### Plasmid construction

The glycoprotein expression plasmids were constructed similarly to previously reported [29]. The cDNA of SFV-G was amplified from the pSFV helper expression vector (a gift from Dr. Robert Chow, University of Southern California). The amplified fragments for the glycoprotein were subcloned into the vesicular stomatitis virus glycoprotein (VSV-G) expression plasmid pVSV-G (Cell Genesys, Foster City, CA). The resulting plasmid was designated pSFV-G (Figure 1). The lentiviral backbone plasmid (FUGW and its derivatives) used in this study have been previously described [31].

### Production of pseudotyped viral particles

Recombinant lentiviruses were prepared by transient transfection of 293T cells using a standard calcium phosphate precipitation protocol [49]. The viral supernatants were harvested 48 and 72 hrs post-transfection and filtered through a 0.45-µm filter. To prepare concentrated viruses, the viral supernatants were ultracentrifugated (Optima L-80K preparative ultracentrifuge, Beckman Coulter) at 50,000×g for 90 min. Mammalian cell-derived viral stocks with high-mannose glycans were generated by transient transfection of 293T cells, which were subsequently cultured in 1 mM 1-deoxymannojirimycin (DMJ, Sigma-Aldrich).

### Confocal imaging of GFP-vpr labeled virions

GFP-vpr-labeled lentiviral particles were produced as previously described [32]. Fresh viral supernatant was overlaid on polylysine-coated coverslips in a 6-well culture dish and centrifuged at 3,700×g at 4°C for 2 hrs using a RT Legend centrifuge. The coverslips were washed with cold PBS twice and incubated with diluted rabbit polyclonal anti-SFV E1/E2 antibody (1∶2000; a gift from Margaret Kielian, Albert Einstein College of Medicine) for 40 min at 4°C. Coverslips were washed with PBS and incubated for 40 min at 4°C with 1∶500 dilutions of secondary antibodies consisting of species-specific Cy5-conjugated anti-immunoglobulin G (Santa Cruz Biotechnology, Santa Cruz, CA). Fluorescent images were acquired by a Zeiss LSM 510 laser scanning confocal microscope with a plan-apochromat oil immersion (63×/1.4) objective.

### Virus attachment assays

Production of [^35^S]-methionine-labeled viruses were produced by transfection of 293T cells as described above. Cells were then depleted of methionine and at 8 hrs post-transfection, [^35^S]-methionine was added to a final concentration of 20 µCi/mL and cells were incubated at 37°C for an additional 12 hrs. [^35^S]-radiolabelled virus was purified from cell supernatants by using a discontinuous sucrose gradient (20%/60% [wt/wt] in TNE buffer [50 mM Tris-HCl, 100 mM NaCl, 1 mM EDTA]), followed by pelleting through 20% sucrose in TNE buffer. Radiolabeled virus particles were resuspended in PBS. Approximately 10^5^ CPM of each radiolabeled virus diluted in PBS was mixed with 10^6^ cells in 1.5 mL microcentrifuge tubes and this mixture was incubated at 4°C for 1 hr with gentle agitation. Cells were washed and ^35^S radioactivity was quantitated with a liquid scintillation counter.

### Determination of titers

To determine viral titer, 2×10^4^ 293T or 293T.DCSIGN cells were transduced with 100 µl of serially diluted viral supernatants with 8 µg/mL of polybrene (Sigma-Aldrich) for 1.5 hrs by spin-inoculation at 2,500 rpm and 25°C using a RT Legend centrifuge. Following the spin-infection, the supernatants were replaced with fresh culture medium and incubated for an additional 48 hrs at 37°C with 5% CO_2_. The GFP expression was measured by flow cytometry. The transduction titer was calculated based on dilution ranges that exhibited a linear response of eGFP expression with viral serial dilution concentration.

### Lentivirus-mediated transduction of cell lines *in vitro*

The 3T3, 3T3-LSIGN and 3T3-DCSIGN cell lines were stained with cross reactive anti-DCSIGN(R) antibody 14E3G7. Target cells (3T3-LSIGN, 3T3-DCSIGN, or 3T3 cells; 0.2×10^5^ per well) were seeded in 96-well culture dishes and spin-infected with viral supernatants (150 µL per well of p24-normalized virus) at 2,500 rpm and 25°C for 90 min using a RT Legend centrifuge. Subsequently, the supernatants were replaced with fresh culture medium and incubated for 48 hrs at 37°C with 5% CO_2_.

### Assays to inhibit pseudotyped virus-mediated infection

In dose-response experiments, 293T.DCSIGN (0.2×10^5^ per well) were incubated with 0.2 to 200 µg/mL of yeast mannan (Sigma-Aldrich) at 37°C for 30 min. SFV-G- or VSV-G-bearing lentiviruses (MOI = 0.8) were incubated for 8 hrs, and then supernatant was replaced with fresh medium. For NH_4_Cl inhibitions, pseudotyped viral particles were spin-inoculated in the presence of increasing concentrations of NH_4_Cl (Sigma-Aldrich) for 90 min at 25°C. 293T.DCSIGN cells were incubated with 5 µg/mL of anti-DCSIGN antibodies (14E3G7, 19F7, DC-28, and isotype control antibody, Santa Cruz Biotechnology), 5 mM EDTA, 15 nM Bafilomycin A_1_, or 10 µg/ml sDC-SIGN, the soluble, tetrameric ectodomain of DC-SIGN produced as previously described [50] at 37°C for 30 min, and then inoculated SFV-G- or VSV-G-bearing lentiviruses at an MOI ∼0.8 for 8 hrs. Similarly, virus was incubated with 25 µg/mL concavalin A (Sigma-Aldrich) for 1 hr at 37C then incubated with 293T.DCSIGN cells for 8 hrs before changing to fresh D10 medium.

### Transduction of human PBMC-derived DCs

Peripheral blood mononuclear cells (PBMC) from two healthy human donors were purchased from AllCells (Emeryville, CA). PBMC were differentiated into DCs as described previously [36]. After two days of culture, DCs were identified by examining the surface markers (CD11C^+^, DC-SIGN^+^) using flow cytometry analysis on cells stained with anti-CD11c antibody and anti-DCSIGN antibody (BD Biosciences). Monocyte-derived DCs (MoDC) on day 2 were exposed to virus at the required MOI based on 293T cells. For inhibition of DC-SIGN-mediated transduction, DCs were incubated with 20 µg/mL of anti-DC-SIGN(R) antibody (14E2G7, Santa Cruz Biotechnology) or 200 µg/mL yeast mannan (Sigma-Aldrich) at 37°C for 30 min and then inoculated with 293T-produced FUGW/SFVG or FUGW/VSVG lentiviruses at an MOI = 10 for 8 hrs before the media was changed.
